# Stage at Diagnosis and Molecular Subtype Distribution of Breast Cancer in Sub‐Saharan Africa: A Systematic Review

**DOI:** 10.1002/cnr2.70594

**Published:** 2026-06-10

**Authors:** Aïssatou Dada Fall, Veronica Veses, Chirag C. Sheth

**Affiliations:** ^1^ Department of Medicine and Surgery Universidad CEU Cardenal Herrera, CEU Universities Alfara del Patriarca Spain; ^2^ Department of Biomedical Sciences Universidad CEU Cardenal Herrera, CEU Universities Alfara del Patriarca Spain

**Keywords:** breast cancer, breast neoplasia, molecular subtype, stage, Sub‐Saharan Africa

## Abstract

**Background:**

This systematic review analyzes the stage at diagnosis of breast cancer and the distribution of molecular subtypes across sub‐Saharan Africa between January 2014 and December 2025. A search on Medline, African Journals Online, Web of Science, and Embase was performed.

**Recent Findings:**

Distribution of molecular subtypes, such as Luminal A or B, HER2‐enriched and Triple‐negative, was also analyzed. From 125 selected studies comprising 42689 patients with staged breast cancer, 63.1% were diagnosed at a late stage, with regional variations (74.5% in Middle Africa, 68.5% in Eastern Africa, 71% in Western Africa, and 55.2% in Southern Africa).Luminal subtypes (A and B) accounted for 68.2% of cases, followed by triple‐negative breast cancer (21.1%) and HER2‐enriched tumors (10.6%).

**Conclusions:**

Our findings provide an updated synthesis of the stage at diagnosis of breast cancer in sub‐Saharan Africa since January 2014. Although a high proportion of patients continue to present at late stage, comparisons with earlier reports should be interpreted cautiously due to heterogeneity in study populations, regional representation, and reporting practices. These findings should be interpreted as an updated regional synthesis rather than evidence of a confirmed temporal improvement in stage at diagnosis.

**Trial Registration:**

The review protocol was not registered in PROSPERO which we acknowledge as a limitation

## 1 Introduction

Since the early 2000s, breast cancer (BC) has remained the most diagnosed cancer in women worldwide. In 2020, over 2.3 million new cases of breast cancer were diagnosed globally, and it is estimated that it resulted in the death of 665 000 patients [1]. The burden of breast cancer is expected to increase in the coming years, with projections estimating a 40% rise in new cases by 2040, resulting in around 3 million new cases annually [2]. Unfortunately, this surge is anticipated to also lead to a 50% increase in the mortality of breast cancer [2]. This would raise the number of deaths related to breast cancer from 685 000 patients to around 1 million patients in 2040 [2].

Incidence rates of breast cancer in Africa have been suffering a pronounced escalation. Despite its lower incidence in Africa compared to other continents (8.3% in 2020), the mortality rate is much higher, accounting for 12.5% of global deaths [3]. By 2040, the incidence of breast cancer in Sub‐Saharan Africa (SSA) is projected to increase by 81.3%, rising from 134 000 (in 2022) to 242 000 new cases [3]. In 2022, the mortality of breast cancer in this region was around 68 000, and it is estimated that it will rise by 82.6%, causing around 124 000 deaths by 2040 [3]. In contrast, the increased mortality might appear disproportionate when compared to the estimated 19.7% rise in mortality rates in Europe [3]. This disproportionate mortality highlights significant disparities in access to early detection, timely diagnosis, and effective treatment between high‐income and low‐resource settings.

The higher mortality of BC in SSA is largely attributable to the high proportion of patients diagnosed at advanced stages. According to a meta‐analysis done by Jedy‐Agba et al., 77% of the patients in Sub‐Saharan Africa were diagnosed at late stages (III‐IV) before 2014 [1]. The elevated mortality rate could also stem from factors such as lack of awareness, education, social barriers, as well as limited access to high quality care in this region and misconceptions about this disease [4]. However, despite increasing attention to early detection strategies in recent years, it remains unclear whether these efforts have translated into measurable changes in stage at diagnosis across the region.

In Sub‐Saharan Africa, there is a common misconception that breast cancer patients are younger compared to the global average. This observation, however, is primarily attributed to this region's younger population demographics rather than a higher prevalence of younger patients being affected by BC. This may also provide insight on the predictions anticipating the rise in incidence of BC in the coming years, as the population continues to age. Nevertheless, the consistently reported younger age at diagnosis raises important clinical and epidemiological questions, particularly regarding the potential contribution of tumor biology, genetic susceptibility, and reproductive factors in this population.

Some studies suggest that in low‐middle income countries, clinical breast examination appears to be more effective for breast cancer screening compared to mammography, which is more prevalent in high income countries. This is due to its lower cost, high accessibility, and less demanding infrastructure requirements [5]. However, the effectiveness of these approaches remains closely dependent on health system organization, community engagement, and timely access to diagnostic confirmation.

There has been an increased emphasis on identifying molecular subtypes of breast cancer to guide the patients' prognosis. In addition to stage at diagnosis, there has been an increased emphasis on identifying molecular subtypes of breast cancer, as these play a key role in determining prognosis and guiding treatment strategies. This is achieved through the identification of hormone receptors such as progesterone, estrogen, and HER‐2/neu within the tumors. The tumors are classified into four main molecular subtypes derived from the expression of those receptors: Luminal A, Luminal B, HER2‐enriched/positive, and Triple negative [6, 7]. The reports about these subtypes in Africa often yield diverse conclusions. While some studies indicate that most breast tumors in African patients were hormone receptor negative (Triple negative) [8], others reported more varied conclusions [9]. This is significant, particularly considering the Triple‐negative tumors tend to have the poorest prognosis as they lack targeted therapy methods [10, 11]. These discrepancies may reflect not only true biological variation across populations but also differences in diagnostic capacity, classification criteria, and access to immunohistochemical testing.

In recent years, there has been an intensified focus on downstaging breast cancer in Africa. This effort consists of implementing strategies to limit the barriers to early detection of breast cancer, particularly when late presentation of the patients is influenced by psychosocial factors or inadequate healthcare systems in the country [12]. Evaluating whether these initiatives have resulted in changes in stage at diagnosis at a population level is essential to inform future public health strategies.

The aim of this systematic review is to provide an updated synthesis of the stage at diagnosis of breast cancer in Sub‐Saharan Africa since January 2014, and to characterize regional patterns in molecular subtypes in relation to clinical presentation. In doing so, we seek to assess whether previously reported patterns of late stage presentation persist, and to better understand the distribution of molecular subtypes across the region. Additionally, we aim to identify the countries reporting molecular subtype data and to explore how subtype distribution relates to stage at diagnosis.

## 2 Methods

### 2.1 Search Strategy

We searched 4 databases with a publication date range from January 2014 to December 2025, following PRISMA 2020 guidelines [13] (Figure S1). The selected databases were Medline, Web of Science, Embase, and African Journal Online. We selected these databases to ensure comprehensive coverage of both global and regional literature on breast cancer.

An initial keyword search using Medical Subject Headings (MeSH) and the following keywords was conducted: “breast cancer”, “breast neoplasm”, “breast carcinoma”, “breast sarcoma”, “breast tumor”, “breast malignancy”, “stage”, “presentation”, “grade”, “clinical features”, “clinical findings”, and “Africa”. Boolean operators (AND/OR) were used to combine terms (full strategy provided in Table S1). The search strategy was adapted for each database. Reference lists of included studies were also screened to identify additional relevant articles. We did not limit the search based on the race of the patients studied in the articles. We included articles that were written in English and French.

### 2.2 Selection Process

We identified and reviewed the articles for this study in two steps. The first step involved a review of the title and abstract of all the articles obtained through the search strategy to determine their eligibility for inclusion in this study.

Inclusion criteria: Studies reporting on women with histologically confirmed breast cancer in SSA; articles reporting data on stage at diagnosis; studies describing outcomes according to recognized staging systems; articles published in English or French between January 2014 and December 2025.

Exclusion criteria: Publications reporting on data from North African countries (Algeria, Egypt, Libya, Morocco, Sudan, Tunisia, Western Sahara); studies lacking detailed information on human breast cancer; articles reporting on cancers other than breast cancer; studies lacking stage at diagnosis data; studies reporting on male breast cancer cases.

### 2.3 Data Extraction

The main data extracted focused on the stage at diagnosis of the breast cancer patients, categorized as stages I, II, III or IV. Additionally, we included articles which described them as early (I‐II) or late (III‐IV) stages. When multiple publications reported analyses from the same cohort (e.g., SABCHO, ABCDO, or Butaro Cancer Center datasets), the study with the largest sample size or most complete staging data was retained for pooled analyses to avoid double counting of patients. Articles which solely reported the histological tumor grade or lacked detailed TNM stage information were excluded. Information regarding the molecular subtype of the tumors (based on immunohistochemistry and Ki‐67 percentage) was also retrieved. When possible, we estimated the different molecular subtypes as Luminal A, Luminal B, HER2‐enriched and Triple negative. Studies reporting incomplete or non‐specific molecular subtype classifications (e.g., reporting only triple‐negative breast cancer or aggregated luminal categories without disaggregation) were excluded from subtype‐specific analyses to ensure comparability across studies. Patients' factors such as the type of study, the country of the study, the race of the patients, the number of patients of each publication, the clinical setting of the studies, the age at diagnosis of the patients, their parity, the menopausal state, the comorbidities of the patients were identified.

### 2.4 Risk of Bias Assessment

Risk of bias in the included studies was assessed using the ROBINS‐I tool (Risk Of Bias In Non‐randomized Studies of Interventions). Each study was evaluated across the following domains: bias due to confounding; bias in selection of participants; bias in classification of interventions; bias due to deviations from intended interventions; bias due to missing data; bias in measurement of outcomes; and bias in selection of the reported result.

The reviewers independently assessed risk of bias, and discrepancies were resolved through discussion. Each domain was judged as low, moderate, serious, critical risk of bias, or no information, according to ROBINS‐I guidance. An overall risk of bias judgment was assigned to each study based on the highest level of risk identified across domains.

Given the observational and predominantly hospital‐based nature of the included studies, many were judged to be at moderate to serious risk of bias, particularly in the domains of confounding and participant selection. A summary of the risk of bias assessments is provided in Table S2.

### 2.5 Publication Bias Assessment

Given the descriptive nature of this review and the substantial heterogeneity in study design, populations, and reported outcomes, formal assessment of publication bias (e.g., funnel plots or Egger's test) was not performed. This is consistent with recommendations for non‐comparative systematic reviews where pooled effect estimates are not generated.

### 2.6 Data Analysis

The main outcome analyzed from the data extracted was the percentage of patients diagnosed with late stage breast cancer (defined as III, III/IV or IV) calculated as the proportion of patients with stage III–IV disease (n_34_/n). Using Excel, we graphically displayed the percentages of late stage diagnoses by regions (Western Africa, Eastern Africa, Southern Africa and Middle Africa) [14] and estimated the distribution of the molecular subtypes in each region. We utilized maps (Mapchart) for visualization, illustrating the represented countries along with the corresponding number of articles attributed to each. Given the heterogeneity in reporting across studies, including variability in the availability of diagnosis‐year data and contextual variables (e.g., urban versus rural setting and country‐level indicators), analyses were conducted using a cross‐sectional synthesis approach. This approach was selected to ensure consistency and comparability across studies while minimizing bias introduced by incomplete temporal or contextual data. Accordingly, formal temporal trend analyses or adjusted meta‐regression models accounting for country‐level factors were not undertaken.

## 3 Results

**FIGURE 1:**
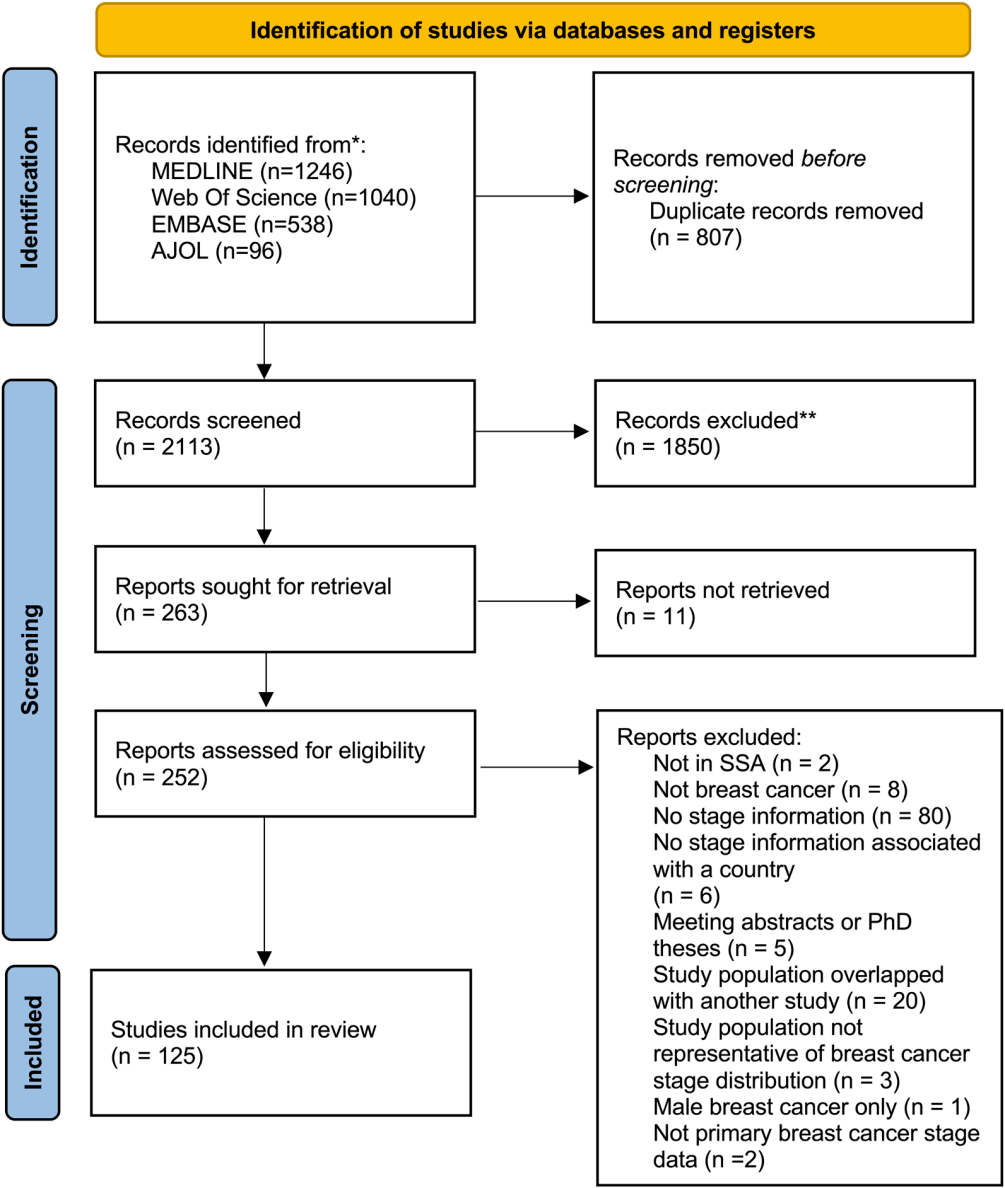
Study selection shown through a PRISMA flowchart [13].

We retrieved 2920 articles using the mentioned search strategy. After the first step of the screening, we assessed 252 full‐text articles and retained 125 articles as relevant to our study (Figure 1). The number of included studies reflects the final eligibility criteria applied after full‐text screening.

**FIGURE 2:**
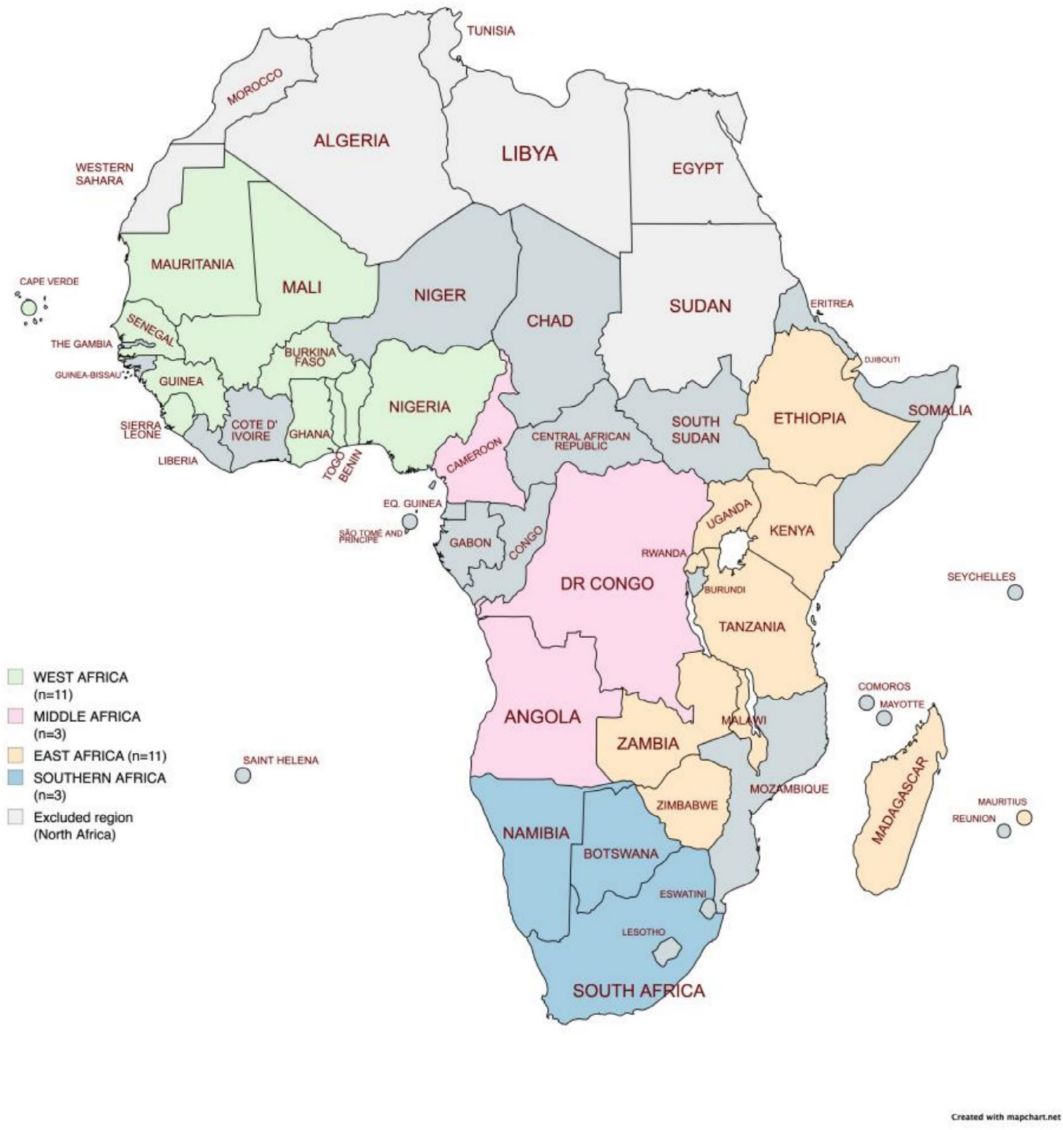
Map representing the origin of the study populations included in this study. Geographical distribution of studies included in the systematic review across Sub‐Saharan Africa. A total of 127 studies were conducted across 28 countries, with the highest representation from Nigeria, Ethiopia, and South Africa, and limited data from Central Africa.

The articles included 28 countries in Sub‐Saharan Africa. Approximately 28.7% of the studies were conducted in Eastern Africa, 46.3% in Southern Africa, 20.8% in Western Africa, and 4.2% in Middle Africa (Figure 2).

One of the included studies [15] consisted in multicenter cohorts' studies that spanned multiple countries. As we were able to identify the relevant stages of breast cancer for each of the countries (Nigeria, Zambia, Namibia, and Uganda), they were also included in the study.

This study comprised 42 689 patients with staged breast cancer (Table 1). The patients were staged using either the Manchester staging system or the American Joint Committee on Cancer breast cancer system (AJCC) and categorized as Stage I, II, I/II, III, IV, and III/IV in the extraction table. The molecular subtypes were based on immunohistochemistry and Ki‐67 percentage.

**TABLE 1:** Demographics and study characteristics.

	Patients with staged breast cancer, *n* (%)	Mean age at diagnosis (years)
Western Africa	8873 (20.8%)	
Benin [16]	284 (0.7%)	47.4
Burkina Faso [17, 18]	393 (1.1%)	47.4
Cape Verde [19]	584 (1.4%)	52.1
Ghana [20, 21, 22, 23, 24, 25, 26, 27, 28]	1915 (4.5%)	49.1
Guinea [29, 30]	168 (0.5%)	48.2
Mali [31]	60 (0.2%)	43.72
Mauritania [32]	540 (1.5%)	39
Nigeria^a^ [12, 15, 33, 34, 35, 36, 37, 38, 39, 40, 41, 42, 43, 44]	3818 (10.9%)	46.4
Senegal [45, 46, 47]	214 (0.6%)	37.3
Sierra Leone [48]	228 (0.5%)	47
Togo [49, 50, 51]	669 (1.9%)	43.2
Eastern Africa	12 255 (28.7%)	
Djibouti [52]	102 (0.3%)	48
Ethiopia [53, 54, 55, 56, 57, 58, 59, 60, 61, 62, 63, 64, 65, 66, 67, 68]	5249 (12.3%)	42.1
Kenya [69, 70, 71, 72, 73, 74, 75, 76]	761 (1.8%)	49
Madagascar [77]	62 (0.1%)	52.83
Malawi [78, 79, 80]	236 (0.6%)	49.1
Mauritius [81]	1059 (2.5%)	NR
Rwanda [82, 83, 84, 85, 86]	1101 (2.6%)	49.4
Uganda^a^ [15, 87, 88, 89]	1191 (2.8%)	45.7
Tanzania [90, 91, 92, 93, 94, 95, 96, 97, 98]	1625 (3.8%)	50.5
Zambia^a^ [15, 99, 100]	577 (1.4%)	NR
Zimbabwe [101, 102]	292 (0.7%)	51.6
Middle Africa	1812 (4.2%)	
Angola [103, 104]	1455 (3.4%)	47
Cameroon [105]	271 (0.6%)	47
Democratic Republic of the Congo [106]	86 (0.2%)	NR
Southern Africa	19 749 (46.3%)	
Botswana [107, 108, 109, 110]	876 (2.1%)	53.2
Namibia^a^ [15]	477 (1.1%)	NR
South Africa [111, 112, 113, 114, 115, 116, 117, 118, 119, 120, 121, 122, 123, 124, 125, 126, 127, 128, 129, 130, 131, 132, 133, 134, 135, 136, 137, 138, 139]	18 396 (43.1%)	52.5
Total	42 689	48

*Note:* Proportion of patients with staged breast cancer in each region of Sub‐Saharan Africa and estimated mean age at diagnosis for each country.

^a^ 1 article [15] was from a multicenter study which spanned across 4 different countries' populations (Namibia, Nigeria, Uganda, and Zambia).

The included publications encompassed retrospective cohorts, prospective cohort studies, cross‐sectional studies, case–control studies, case series, randomized controlled trials, and qualitative studies.

### 3.1 Stages at Diagnosis of BC

Of the 42 689 patients included in this study, 26 949 (63.1%) were diagnosed with late stage disease (stage III–IV), while 12 124 (34.7%) were diagnosed at early stage.

**FIGURE 3:**
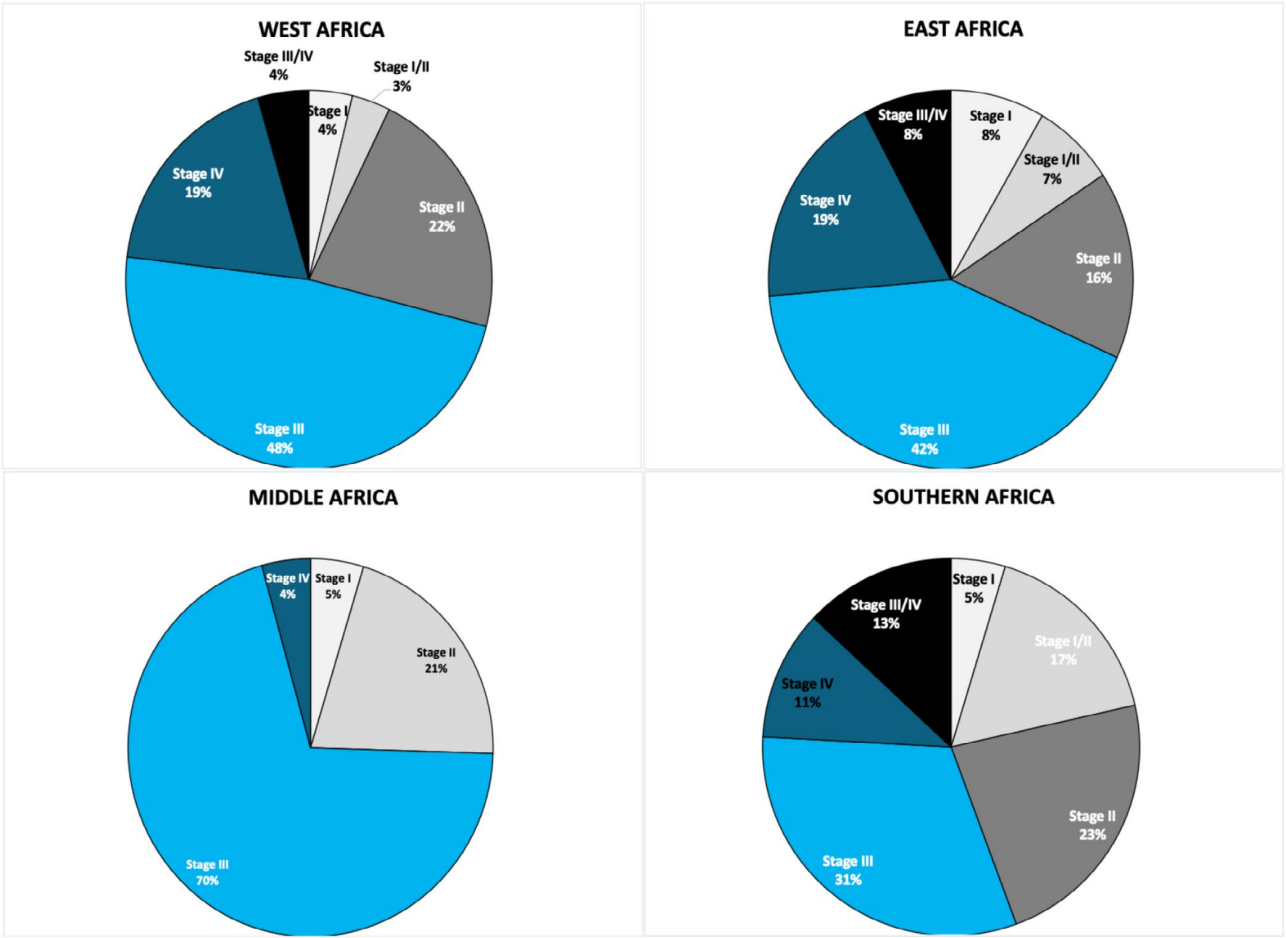
Proportion of breast cancer stages in each region of Sub‐Saharan Africa.

**FIGURE 4:**
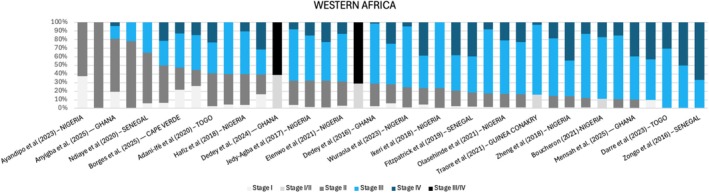
Study specific stage distribution of breast cancer in Western Africa. This chart shows the proportion of each stage of breast cancer in 38 articles studying Western African populations.

In Western Africa, 8873 patients (20.8% of the total cohort) had reported staging data. Among these, 71% (*n* = 6300) were diagnosed with late stage disease, including 47.9% stage III, 18.6% stage IV, and 4.5% stage III/IV. Early‐stage disease accounted for 29.0% of cases, including 3.8% stage I, 21.8% stage II, and 3.4% stage I/II (Figure 3). The breakdown of breast cancer stage by region, study and country for Western Africa can be found in Figure 4.

**FIGURE 5:**
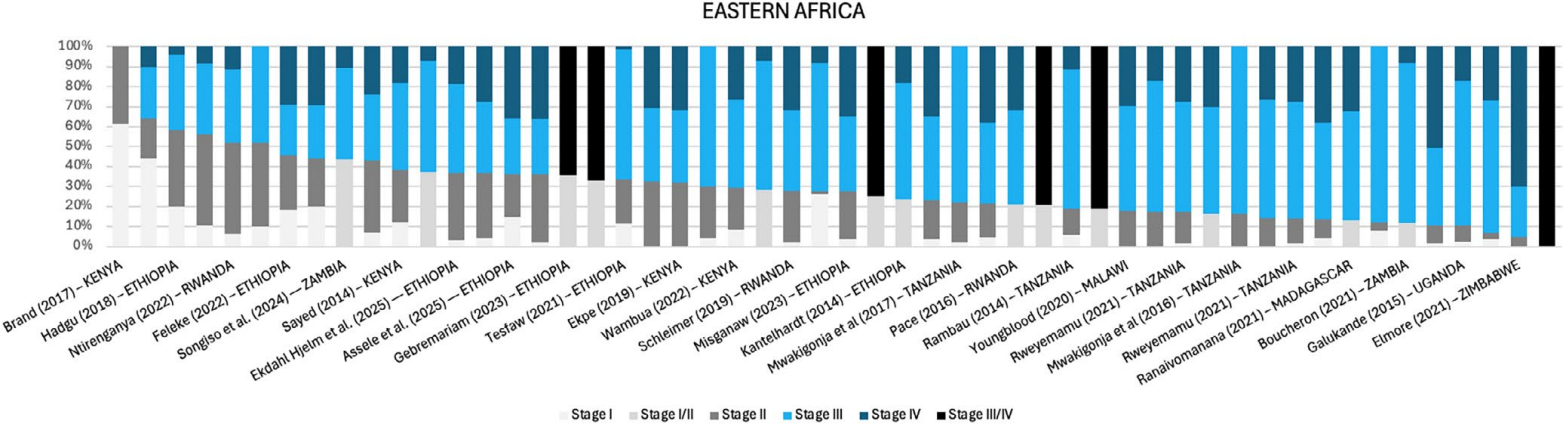
Study specific stage distribution of breast cancer in Eastern Africa. This chart shows the proportion of each stage of breast cancer in 52 study populations in Eastern Africa.

In Eastern Africa, 12 255 patients (28.7% of the total cohort) had reported staging data. Among these, 8391 (68.5%) were diagnosed with late‐stage disease, including 42.4% stage III, 18.5% stage IV, and 7.6% stage III/IV. Early‐stage disease accounted for 31.5% of cases, including 8.1% stage I, 15.5% stage II, and 7.9% stage I/II (Figure 3). The breakdown of breast cancer stage by region, study and country for Eastern Africa can be found in Figure 5.

**FIGURE 6:**
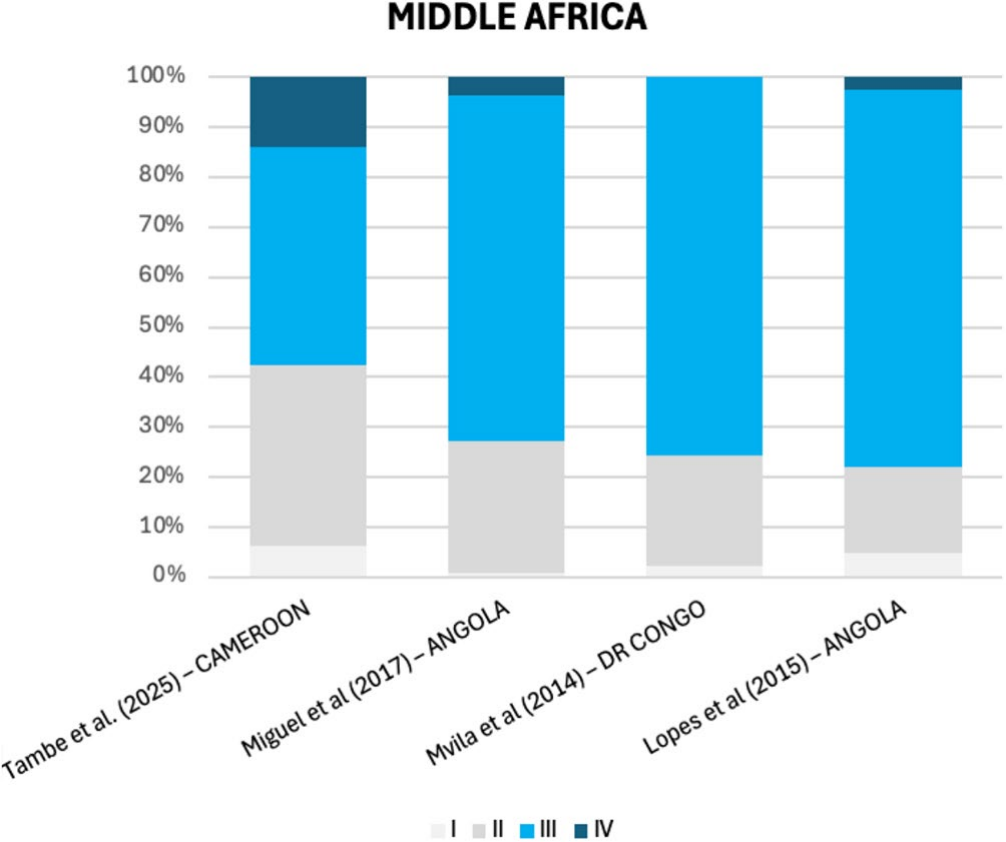
Study specific stage at diagnosis of breast cancer in Middle Africa. This figure shows the proportion of each stage of breast cancer in 4 articles studying Middle Africa.

In Middle Africa, 1812 patients (4.2% of the total cohort) had reported staging data. Among these, 1350 (74.5%) were diagnosed with late‐stage disease, including 70.2% stage III and 4.3% stage IV. Early‐stage disease accounted for 25.5% of cases, including 4.7% stage I and 20.8% stage II (Figure 3). The breakdown of breast cancer stage by region, study and country for Middle Africa can be found in Figure 6.

**FIGURE 7:**
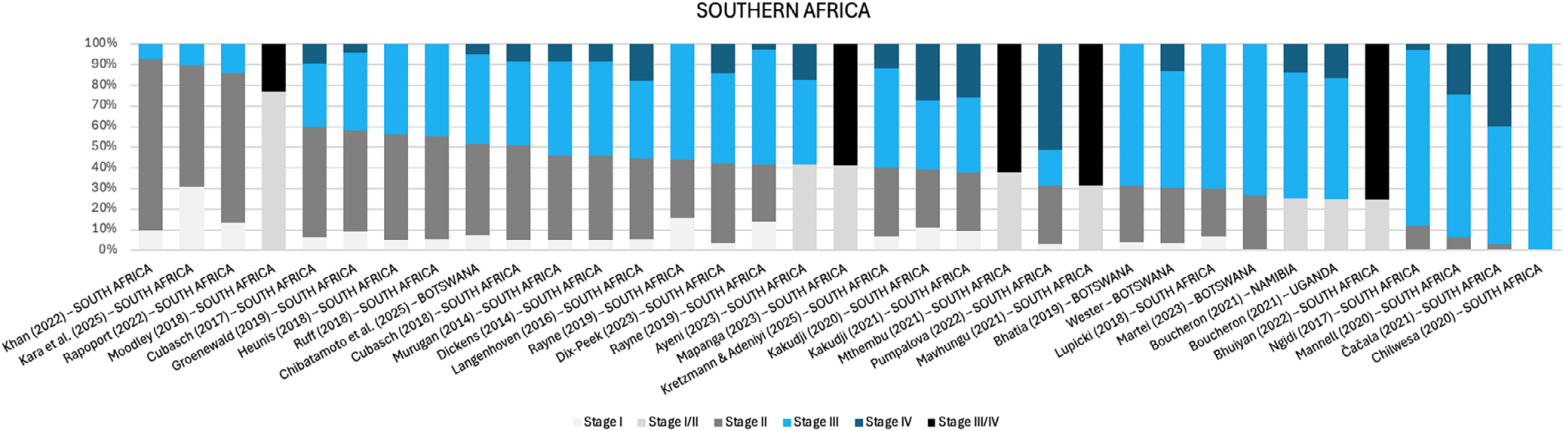
Study specific stage at diagnosis of breast cancer in Southern Africa. This chart shows the proportion of each stage of breast cancer in 35 study populations from Southern Africa.

In Southern Africa, 19 749 patients (46.3% of the total cohort) had reported staging data. Among these, 10 908 (55.2%) were diagnosed with late‐stage disease, including 31.0% stage III, 11.0% stage IV, and 13.2% stage III/IV. Early‐stage disease accounted for 44.8% of cases, including 4.7% stage I, 23.4% stage II, and 16.6% stage I/II (Figure 3). The breakdown of breast cancer stage by region, study, and country for Southern Africa can be found in Figure 7.

### 3.2 Molecular Subtype Analysis

**FIGURE 8:**
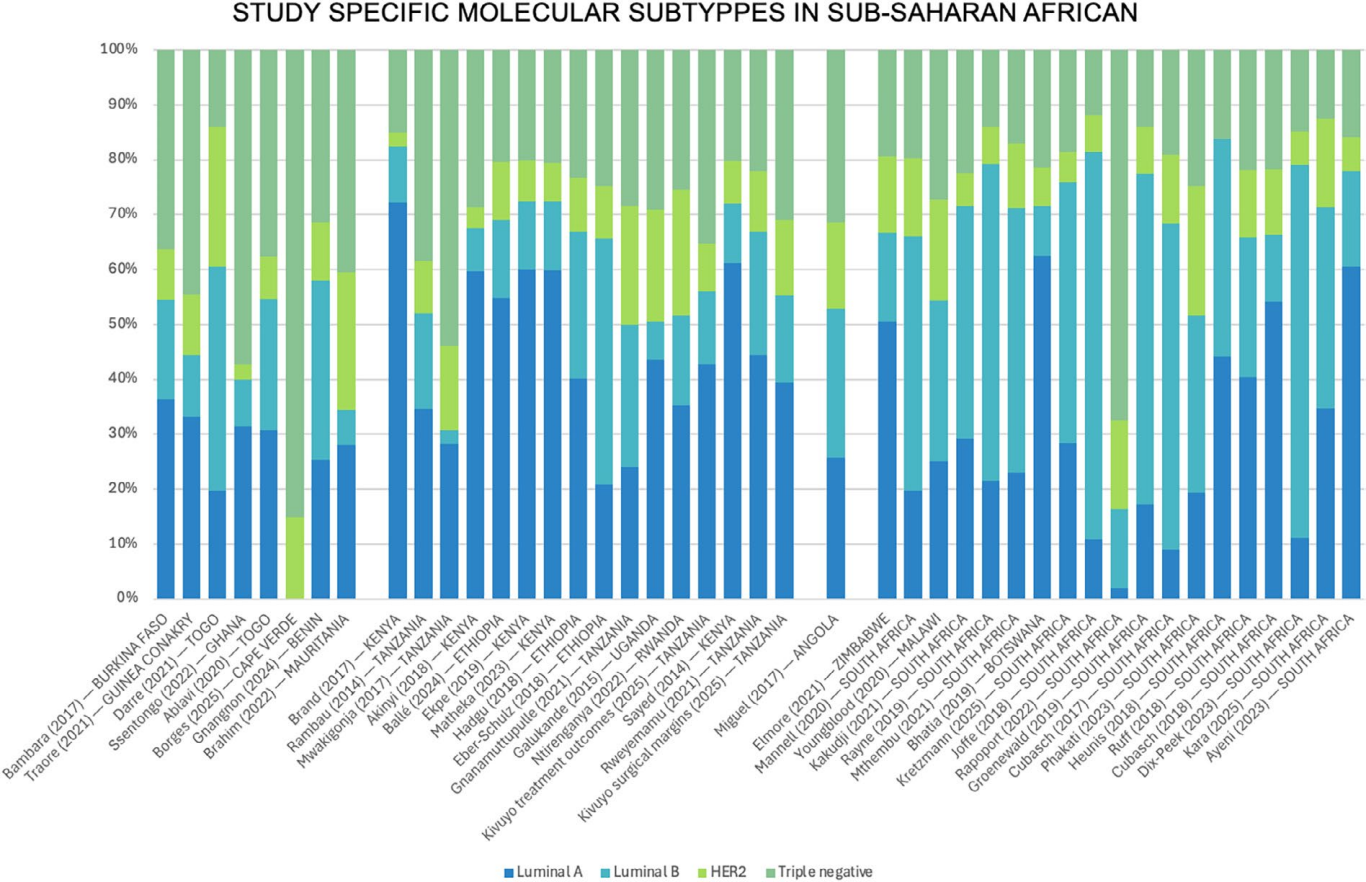
Study specific molecular subtyping of breast cancer in Sub‐Saharan Africa. This chart regroups the proportions of each molecular subtype of breast cancer found in 41 articles.

Molecular subtype distribution was reported in a subset of included studies with sufficient detail to allow classification into luminal A, luminal B, HER2‐enriched, and triple‐negative breast cancer (TNBC). The breakdown of molecular subtype by study and country can be found in Figure 8.

Across all included studies, a total of 13 621 tumors had available molecular subtype data. Luminal A was reported in 5058 cases (37.1%), followed by luminal B in 4240 cases (31.1%). Together, luminal subtypes accounted for 68.2% of all cases. Triple‐negative breast cancer represented 2877 cases (21.1%), while HER2‐enriched tumors accounted for 1446 cases (10.6%).

**FIGURE 9:**
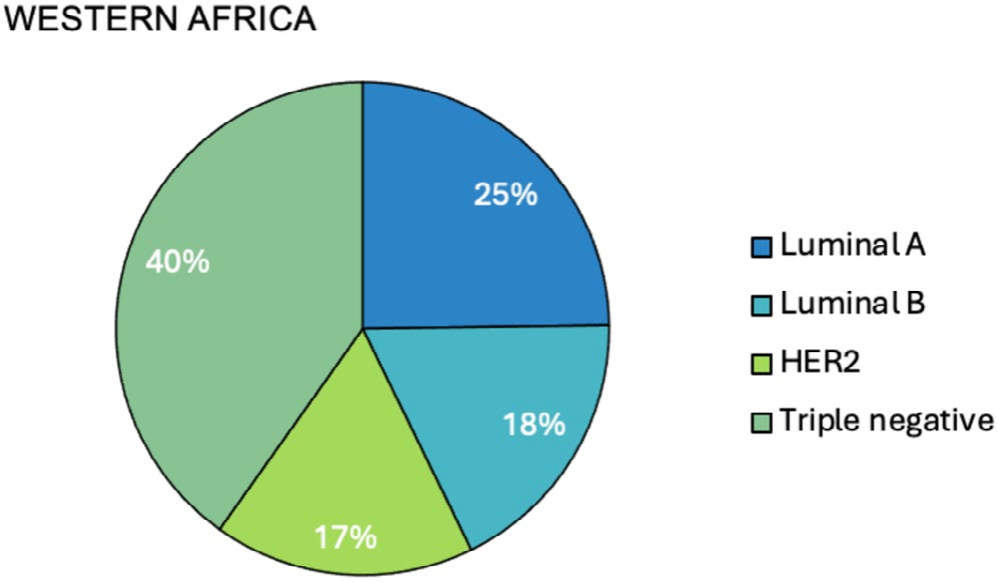
Distribution of molecular subtypes across Western Africa.

In Western Africa, TNBC was the predominant subtype, accounting for 40.1% of cases, followed by luminal A (24.8%), luminal B (17.9%), and HER2‐enriched tumors (17.1%) (Figure 9).

**FIGURE 10:**
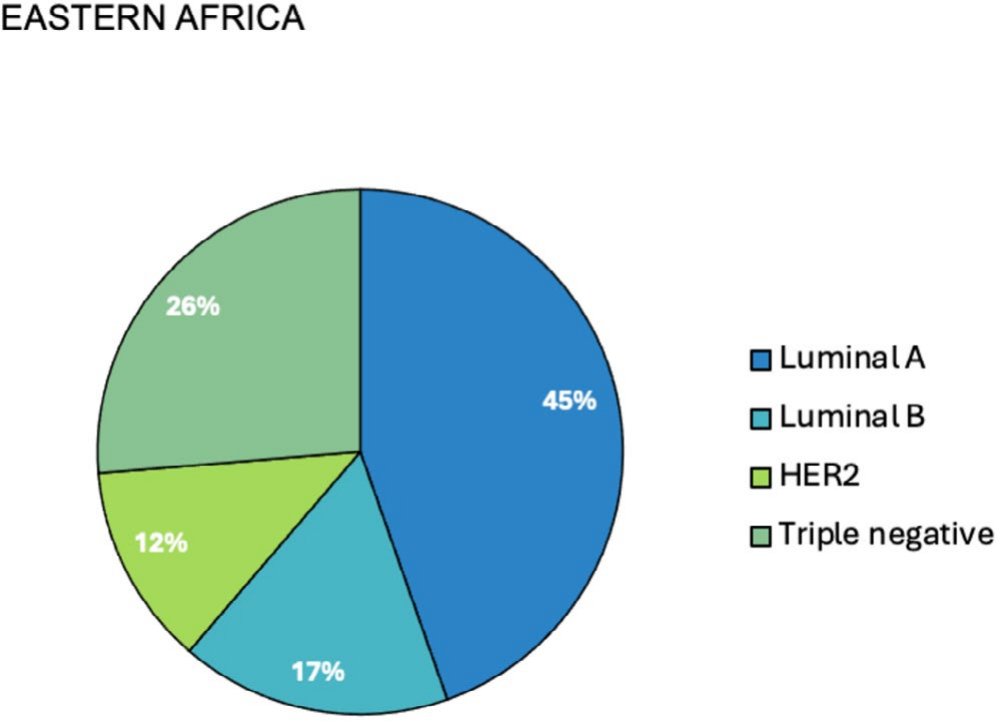
Distribution of molecular subtypes across Eastern Africa.

In Eastern Africa, luminal A was the most frequent subtype (44.4%), followed by TNBC (26.5%), luminal B (16.7%), and HER2‐enriched tumors (12.4%) (Figure 10).

**FIGURE 11:**
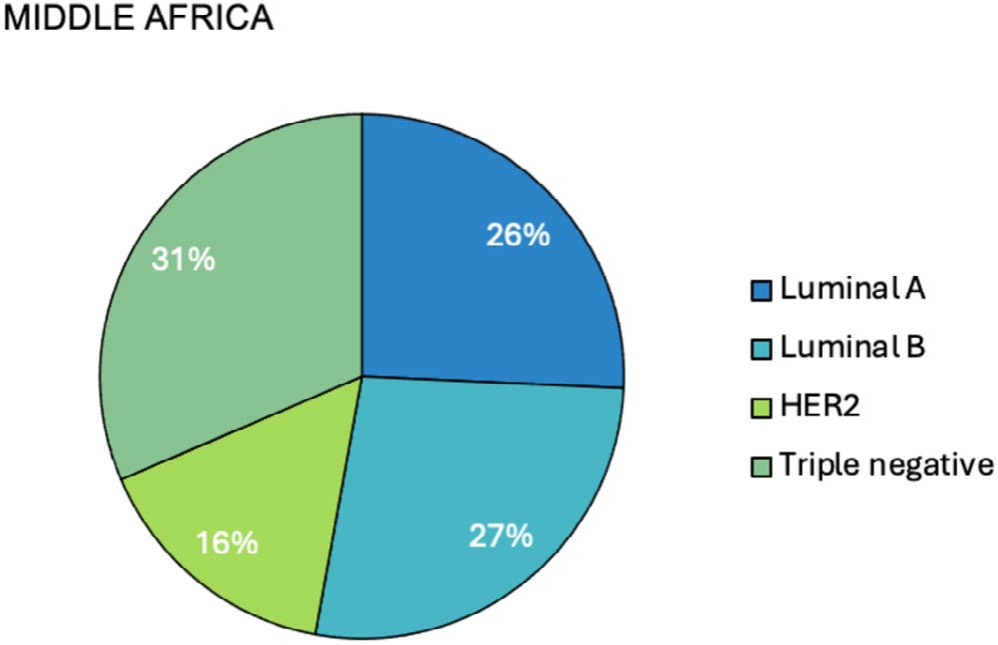
Distribution of molecular subtypes across Middle Africa.

In Middle Africa, based on limited data from a single study, TNBC accounted for 31.4%, luminal B for 27.1%, luminal A for 25.7%, and HER2‐enriched tumors for 15.7% (Figure 11).

**FIGURE 12:**
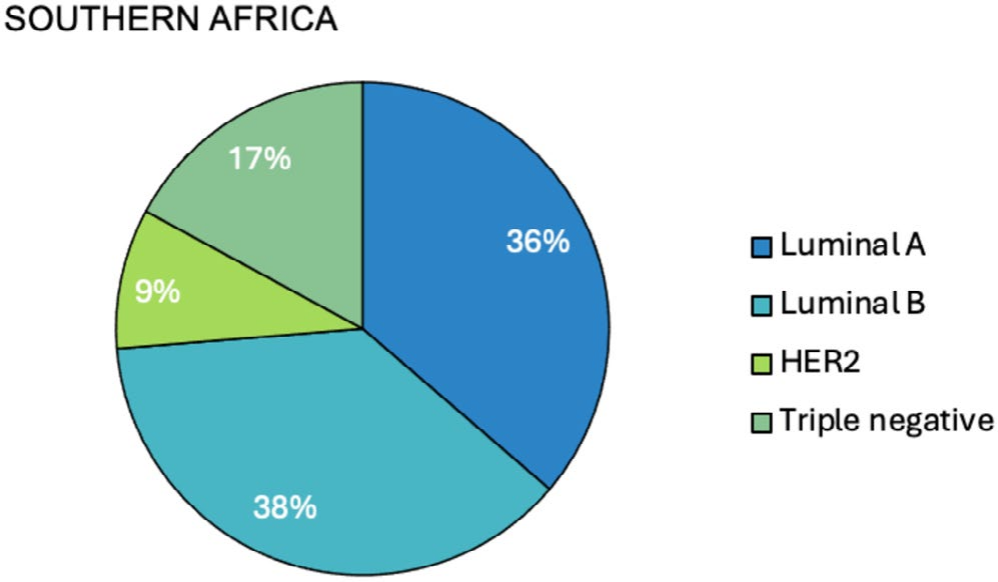
Distribution of molecular subtypes across Southern Africa.

In Southern Africa, luminal subtypes predominated, with luminal B (37.2%) and luminal A (36.4%) representing the majority of cases. TNBC accounted for 17.1%, while HER2‐enriched tumors represented 9.2% (Figure 12).

**FIGURE 13:**
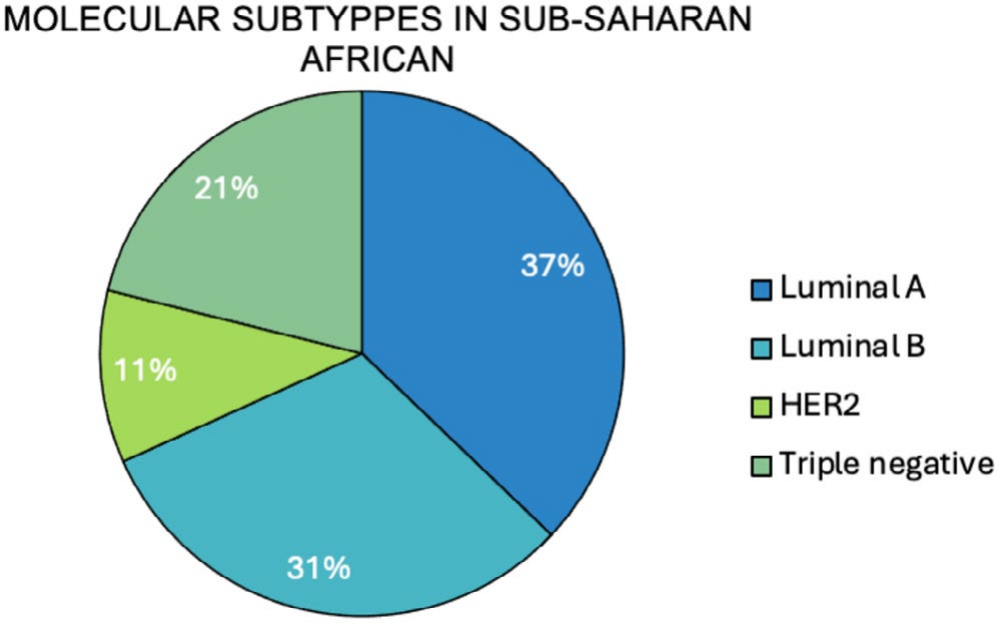
Distribution of molecular subtypes across Sub‐Saharan Africa.

Overall, these findings demonstrate substantial regional variation in molecular subtype distribution, with a higher proportion of TNBC in Western Africa and a predominance of luminal subtypes in Southern Africa. The global distribution of molecular sub‐types of breast cancer throughout the Sub‐Saharan region is shown in Figure 13. These results should be interpreted with caution given heterogeneity in study design, variability in immunohistochemistry availability, and differences in subtype classification across studies.

## 4 Discussion

### 4.1 Main Findings

This review synthesized evidence on the stage at diagnosis and molecular subtypes of BC in SSA between January 2014 and December 2025. The study examined 125 articles which reported on the stage at diagnosis of breast cancer. A subset of these studies also reported molecular subtype data. The study‐specific distribution of the molecular subtypes in each region of SSA is represented in Table S3. The included studies were distributed across 28 countries, with a disproportionate representation from Nigeria, Ethiopia, and South Africa, while several regions, particularly Central Africa, remained underrepresented. We found that the majority of patients (63.1%) continue to present at late stage (III‐IV). These estimates are broadly comparable to earlier reports [140]; however, direct comparisons should be interpreted with caution due to heterogeneity in study populations, reporting practices, and country representation. Substantial regional variation was observed: late‐stage presentation was most frequent in Middle and Eastern Africa and relatively less common in Southern Africa (Figure 3). Regarding the molecular subtypes, Luminal A and B tumors were most frequent overall (Figure 13), but triple‐negative breast cancer was disproportionately higher in West and East Africa (Figures 9, 10) [9, 48]. These findings highlight encouraging trends and persistent challenges in breast cancer detection and management across the region. These results should be interpreted as a contemporary regional synthesis rather than a direct temporal comparison, given the heterogeneity of included studies.

### 4.2 Age at Diagnosis, Interpretation and Implications

The mean age at diagnosis reported across included studies was approximately 48 years (Table S3), consistent with previous reports indicating that breast cancer in Sub‐Saharan Africa is frequently diagnosed between 35 and 49 years of age [140]. This finding has important epidemiological and clinical implications.

However, the interpretation of a younger age at diagnosis requires careful contextualization. The population structure in Sub‐Saharan Africa is substantially younger than in high‐income countries, which contributes to a lower median age at cancer diagnosis at the population level [3]. In addition, the majority of included studies were hospital‐based, which may introduce selection bias, as younger patients may be more likely to access tertiary care or be referred for specialized treatment.

We extracted additional clinical variables, including parity, menopausal status, and comorbidities, where available. However, reporting of these variables was highly inconsistent across studies, precluding a robust quantitative synthesis. When reported, several studies suggested higher parity, premenopausal status, and variable comorbidity profiles, although these findings were not consistently documented across regions [12]. These factors are relevant, as reproductive patterns (e.g., age at first childbirth, parity), hormonal status, and comorbidities have been implicated in breast cancer risk and subtype distribution [4, 12]. Nevertheless, the heterogeneity and limited reporting of these variables in the included studies restrict the ability to draw firm conclusions regarding their contribution to the observed age distribution.

From a clinical perspective, a relatively younger age at diagnosis of BC has important implications. Breast cancer in younger women is often associated with more aggressive disease, including a higher likelihood of triple‐negative subtypes, and may require tailored management strategies, including considerations related to fertility preservation, long‐term survivorship, and psychosocial support [10, 11].

Overall, these findings highlight the need for improved and standardized reporting of patient‐level clinical variables in studies from Sub‐Saharan Africa, which would enable a more comprehensive understanding of the determinants of age at diagnosis and inform targeted prevention and management strategies.

Importantly, these findings should not be interpreted as evidence of an inherently earlier biological onset of breast cancer in this population, but rather as the result of complex interactions between demographic structure, healthcare access, and potential biological differences.

### 4.3 Trends in Stage at Diagnosis, Progress and Gaps

Compared with older literature suggesting late‐stage presentation in around 77%–80% of women in Sub‐Saharan Africa [140], our estimates indicate that approximately two‐thirds of patients are diagnosed at stage III or IV. However, these findings should not be interpreted as evidence of a temporal improvement. The included studies were highly heterogeneous with respect to country representation, study design, and clinical setting, which limits direct comparability with earlier reports.

In addition, differences in the composition of included studies, including variation in country‐level healthcare systems, urban versus rural settings, and access to diagnostic services, may influence aggregate estimates. Therefore, comparisons with prior literature should be interpreted with caution, as they may reflect differences in study populations rather than true changes over time. Despite these limitations, the findings confirm that late‐stage presentation remains highly prevalent across Sub‐Saharan Africa, underscoring the need for improved early detection, timely diagnosis, and access to care.

While improvements in awareness and diagnostic capacity may have contributed to changes in stage at presentation in some settings, robust longitudinal data are lacking, limiting the ability to confirm true temporal trends. In addition, variations in staging classification, diagnostic pathways, and reporting standards across studies may further limit comparability between datasets [112, 140]. The potential impact of the COVID‐19 pandemic on stage at diagnosis should also be considered when interpreting these findings. Disruptions to screening, diagnostic pathways, and treatment services have been reported globally, including in Sub‐Saharan Africa. However, the included studies did not provide sufficiently granular or standardized reporting of diagnosis periods to enable a robust assessment of COVID‐related effects. As such, the present analysis does not attempt to quantify this impact, and future studies with temporally resolved data will be essential to evaluate the influence of the pandemic on stage at diagnosis in this setting.

Overall, these findings are best interpreted as an updated regional synthesis rather than a formal assessment of temporal trends, highlighting both the persistence of late‐stage presentation and the need for standardized, longitudinal data across the region.

### 4.4 Regional Differences in Stages and Molecular Subtypes

Marked regional heterogeneity was observed in both stage at diagnosis and molecular subtype distribution across Sub‐Saharan Africa.

In our study, we found that 40% of the patients from the Western African region were diagnosed with Triple‐negative breast cancer (TNBC) as reported in Figure 9. These findings align with another study where 42.36% of the patients from West Africa were diagnosed with Triple‐negative breast cancer [9]. This consistency suggests a higher prevalence of TNBC in this region. However, differences in diagnostic practices and subtype classification methods across studies should be considered when interpreting these findings. Given that Triple‐negative tumors tend to have a worse prognosis and recur more compared to the other subtypes, this is significant [11, 141]. As we've observed in our study, 73% of tumors in West Africa were diagnosed at a late stage (III or IV) (Figure 4). The combination of high TNBC fraction and a high proportion of late‐stage presentation likely contributes to the region's disproportionate mortality burden.

Most of the patients in Eastern Africa (68.5%) were also found in the late‐stage at diagnosis group (Figure 5). A publication by Magwesela et al. [142] helped identify multiple barriers to BC screening in East African women. They surveyed patients on their attitudes towards breast cancer screening. While some women were favorable towards screening due to its potential benefits such as early detection, increased survival, and decreased treatment costs, another group perceived no advantage. Those women were concerned with the fact that even if they were diagnosed earlier, they might lack the financial resources to afford treatments. They feared that screening could potentially cause them more harm.

Understanding the perspectives of the patients is essential to help control this cancer. These findings highlight the critical role of health system trust, financial accessibility, and patient education in influencing early presentation. These perceptions, along with various other barriers, contribute to the late‐stage diagnosis and increasing mortality of BC in SSA [143, 144]. Additionally, a publication suggested that the aggressive nature of breast cancer in East Africa might be related to a high genetic risk factor. They concluded that 6% of their study population were BRCA1/2 pathogenic variant carriers, and that they were more likely to be found with Triple‐negative breast cancer [145]. This finding may also apply to other regions in Sub‐Saharan Africa. There is currently a lack of genetic studies in Sub‐Saharan Africa due to the high cost and infrastructure requirements. The implementation of genetic tests in SSA would facilitate personalized treatments and help reduce the mortality of breast cancer [145, 146].

Based on our analysis, in Eastern Africa, the Luminal A subtype was the most common (44%), followed by the Triple negative breast cancer (26,5%) (Figure 10). Our results were comparable to those of a study realized by Popli et al. [147] which found that 48% of the tumors were Luminal A in East Africa. In contrast, in the United States, where Luminal subtypes are predominant, only 12.2% of patients are diagnosed with TNBC [148].

In this study, a limited number of articles provided data on the stage at diagnosis of BC in Middle Africa (Figure 6), likely due to lack of screening, limited infrastructure, and awareness of the population. In a previous study conducted by Jedy Agba and colleagues [140], published in 2016, no articles were identified from this region. We were able to determine that 74.5% of patients in this region were diagnosed with late‐stage breast cancer. The limited geographic representation underscores the need for improved cancer registries and research capacity in this region. The paucity of studies from this region prevents confident generalization, but the available data point to profound gaps in diagnostic and registry capacity.

When considering the molecular subtypes, only one article from the Middle African region was included in our analysis (Figure 8). This study included 140 patients from Angola with a known molecular subtype. Due to this small sample size, our results may not accurately reflect the true distribution of molecular subtypes in this region. In Angola, we found that 31% of the tumors were Triple‐negative, followed by the Luminal A and B tumors (26% and 27% respectively) and the HER2‐enriched subtype (16%) (Figure 11). Another recent study, which also only had a small sample size of 302 patients, aimed to estimate the proportion of Triple‐negative breast cancer in Middle Africa. They found that approximately 18.25% of the tumors were Triple‐negative, 8% HER2‐enriched, and 81% Luminal [9].

We must emphasize the need for more studies, including data on breast cancer stage at diagnosis and molecular subtypes, to be published in this region. Only three out of the ten countries in this region were included in this study (Angola, Cameroon, and Democratic Republic of the Congo).

In the previous years, studies providing information on the stage at diagnosis in the Southern Africa region were primarily from South Africa. In our study, most of the patients were also found in South Africa (46%), with some patients identified in Botswana and Namibia as well (Table S3). We observed that 57% of the patients were diagnosed at a late stage in Southern Africa (Figure 3). A publication by Mapanga et al. focusing on South Africa reported that 59% of the patients in South Africa were diagnosed with late‐stage BC [136]. A previous study done by McCormack et al. suggested a decrease in the frequency of late‐stage BC in South Africa to 46% in 2013 [149]. Therefore, the increase in stage III/IV tumors, although coherent with the aging population, may seem alarming. Our results for this region are likely more representative of the South African population, as they represented 93% of the patients from the Southern Africa region. There is a need for additional studies in other Southern African countries, to provide insight into the stages of breast cancer diagnosis and subtypes among their populations.

The most prevalent molecular subtype in Southern Africa was Luminal B (37.1%) and Luminal A (36.3%) as shown in Figure 12. This finding was also confirmed by Onyia et al. in their study, where 67.6% patients in South Africa were found to belong to the Luminal subgroup. Only 17% of the tumors were of the Triple‐negative subtype. Luminal tumors tend to have a more favorable outcome and fewer metastases than the other subtypes [9]. The mortality of breast cancer in Southern Africa in 2022 was around 5693 patients, which was much lower than in the other Sub‐Saharan African regions such as Western Africa, where around 27 901 patients died [1]. This difference might be explained by the higher prevalence of Triple‐negative subtypes in the other Sub‐Saharan African regions. However, differences in healthcare infrastructure, access to treatment, and screening programs likely also contribute to these regional disparities.

The distribution of molecular subtypes varied across regions, with a consistently high burden of triple‐negative breast cancer (TNBC), particularly in West Africa (Figure 8). TNBC proportions were frequently higher than those reported in high‐income settings, where luminal subtypes predominate [10, 140]. In contrast, studies from Southern Africa demonstrated a greater representation of luminal tumors, particularly luminal B, alongside substantial TNBC proportions (Figures 8, 12).

These differences may reflect a combination of factors, including population demographics, genetic and ancestral influences, reproductive patterns, and variability in access to diagnostic tools such as immunohistochemistry [4, 12]. In particular, limited access to standardized pathology services in some settings may contribute to under‐classification or misclassification of subtypes.

The relatively high burden of TNBC has important clinical implications, as this subtype is associated with more aggressive disease, limited targeted treatment options, and poorer outcomes [10, 11]. These findings underscore the need for improved access to accurate diagnostic services and expanded treatment options across the region.

Overall, these findings emphasize that both biological and structural factors must be considered when interpreting regional differences in breast cancer outcomes in Sub‐Saharan Africa.

### 4.5 Strengths and Limitations

The strengths of this study revolve around the large sample size (42689patients), a comprehensive search across multiple databases and inclusion of non‐English publications. However, several limitations should be noted. We prioritized inclusion of studies reporting stage at diagnosis; however, molecular subtype data were inconsistently reported across studies, limiting the completeness of subtype analyses. Additionally, variability in staging definitions, diagnostic methods, and subtype classification across studies may have introduced measurement bias.

Most studies were hospital based, limiting generalizability. Despite these limitations, this study provides one of the most comprehensive and up‐to‐date syntheses of breast cancer stage at diagnosis and molecular subtypes in Sub‐Saharan Africa.

## 5 Conclusion

Compared with earlier reports suggesting that approximately 77%–80% of women in Sub‐Saharan Africa were diagnosed at late stages, our findings indicate that the majority of patients (63.1%) continue to present with stage III or IV disease. However, these findings should be interpreted cautiously and do not constitute evidence of a true temporal improvement, given the substantial heterogeneity in study design, country representation, and clinical settings across the included studies.

Molecular subtype distributions also demonstrated marked regional variability, with a higher prevalence of triple‐negative breast cancer observed in Western and Eastern Africa, and a predominance of luminal tumors in Southern Africa. These differences may contribute to regional disparities in outcomes; however, they should be interpreted with caution due to variability in immunohistochemical assessment, differences in classification criteria, and inconsistent reporting across studies.

Despite these limitations, this review highlights the persistent challenges in early detection and subtype‐specific management of breast cancer in Sub‐Saharan Africa. The high proportion of late‐stage diagnosis underscores ongoing gaps in access to timely diagnosis and care.

Future research should prioritize the development of robust cancer registries and population‐based studies to provide more representative data across the region. Greater attention should also be given to underrepresented areas, particularly Middle Africa, where data remain scarce. In addition, expanding access to molecular and genomic profiling will be essential to better characterize tumor biology and guide tailored prevention and treatment strategies.

Addressing these gaps will be critical to informing targeted interventions aimed at improving outcomes for women with breast cancer across Sub‐Saharan Africa. Efforts to reduce the stage at diagnosis must be accompanied by strengthened health systems, improved access to treatment, and sustained support for patients following diagnosis.

## Author Contributions

**Veronica Veses:** data curation, investigation, validation, writing – review and editing. **Chirag C. Sheth:** conceptualization, data curation, investigation, validation, writing – review and editing. **Aïssatou Dada Fall:** conceptualization, data curation, formal analysis, investigation, validation, writing – original draft, writing – review and editing.

## Funding

The authors have nothing to report.

## Conflicts of Interest

The authors declare no conflicts of interest.

## Supporting information


**Figure S1:** PRISMA checklist (2020) for systematic reviews.

**Table S1:** Search strategy using Boolean operators. The search strategy combined controlled vocabulary (e.g., MeSH terms) and free‐text terms related to breast cancer, stage diagnosis, and Sub‐Saharan Africa. The strategy was adapted for each database.

**Table S2:** Risk of bias assessment of included studies using ROBINS‐I.

**Table S3:** Characteristics of the studies included in this study (country, study design, study period, stages of breast cancer reported and age).

## Data Availability

The data that support the findings of this study are available from the corresponding author upon reasonable request.
