# Supplementary material for: Stage at Diagnosis and Molecular Subtype Distribution of Breast Cancer in Sub‐Saharan Africa: A Systematic Review
Source: Cancer Rep (Hoboken). 2026 Jun 10;9(6):e70594. doi: 10.1002/cnr2.70594 (PMC13250637; doi:10.1002/cnr2.70594)
Supplement: Supplementary file 2 — Table S1: Search strategy using Boolean operators. The search strategy combined controlled vocabulary (e.g., MeSH terms) and free‐text terms related to breast cancer, stage diagnosis, and Sub‐Saharan Africa. The strategy was adapted for each database. [file CNR2-9-e70594-s001.pdf]

Supplementary Table 1: Search strategy using Boolean operators

The search strategy combined controlled vocabulary (e.g., MeSH terms) and free-text terms related to breast cancer, stage at diagnosis, and Sub-Saharan Africa. The strategy was adapted for each database.

**Search strategy**
("breast cancer" OR "breast neoplasm" OR "breast carcinoma" OR "breast tumor" OR<br>"breast tumour" OR "breast malignancy" OR "mammary cancer")
**AND**
("stage" OR "stage at diagnosis" OR "cancer stage" OR "tumor stage" OR "tumour stage" OR<br>"clinical stage" OR "stage distribution" OR "disease stage" OR "advanced stage" OR "late<br>stage" OR "stage III" OR "stage IV")
**AND**
("Africa" OR "sub-Saharan Africa" OR "SSA" OR "East Africa" OR "West Africa" OR "Southern Africa" OR "Central Africa")
