# Supplementary material for: Stage at Diagnosis and Molecular Subtype Distribution of Breast Cancer in Sub‐Saharan Africa: A Systematic Review
Source: Cancer Rep (Hoboken). 2026 Jun 10;9(6):e70594. doi: 10.1002/cnr2.70594 (PMC13250637; doi:10.1002/cnr2.70594)
Supplement: Supplementary file 3 — Table S2: Risk of bias assessment of included studies using ROBINS‐I. [file CNR2-9-e70594-s004.pdf]

Reference	D1 Confounding	D2 Selection of participants	D3 Classification of exposure	D4 Deviations from intended exposure	D5 Missing data	D6 Outcome measurement	D7 Selection of reported result	Overall
Achan (2023)	Serious	Moderate	Moderate	Low	Moderate	Moderate	Moderate	Serious
Adeoluwa et al. (2020)	Serious	Serious	Low	Low	Moderate	Moderate	Moderate	Serious
Akingbade et al. (2022)	Serious	Serious	Moderate	Low	Moderate	Moderate	Moderate	Serious
Akinyi et al (2019)	Serious	Moderate	Low	Low	Moderate	Low	Moderate	Serious
Ali-Gombe et al. (2021)	Moderate	Serious	Low	Low	Moderate	Low	Moderate	Serious
Ayandipo et al. (2023)	Serious	Serious	Moderate	Low	Moderate	Moderate	Moderate	Serious
Ayeni (2023)	Moderate	Moderate	Low	Moderate	Moderate	Low	Moderate	Moderate
Bacha (2021)	Serious	Moderate	Low	Low	Serious	Moderate	Moderate	Serious
Bambara et al. (2017)	Serious	Serious	Low	Low	Moderate	Moderate	Moderate	Serious
Bhatia (2019)	Serious	Moderate	Moderate	Low	Moderate	Moderate	Moderate	Serious
Bhuiyan (2022)	Serious	Moderate	Low	Low	Moderate	Moderate	Moderate	Serious
Boucheron (2021)	Moderate	Moderate	Moderate	Low	Moderate	Low	Moderate	Moderate
Brahim et al. (2022)	Serious	Moderate	Low	Low	Moderate	Low	Moderate	Serious
Brand (2017)	Serious	Moderate	Low	Low	Moderate	Low	Moderate	Serious
Čačala (2021)	Serious	Moderate	Moderate	Moderate	Serious	Moderate	Moderate	Serious
Chilwesa (2020)	Moderate	Moderate	Low	Low	Moderate	Moderate	Moderate	Moderate
Cubasch (2017)	Serious	Moderate	Moderate	Low	Moderate	Moderate	Moderate	Serious
Cubasch (2018)	Moderate	Moderate	Moderate	Low	Serious	Moderate	Moderate	Serious
Darre et al. (2021)	Serious	Serious	Moderate	Low	Moderate	Moderate	Moderate	Serious
Darre et al. (2023)	Moderate	Serious	Low	Low	Moderate	Moderate	Moderate	Serious
Dedey et al. (2016)	Serious	Serious	Moderate	Low	Moderate	Moderate	Moderate	Serious
Diakite et al. (2020)	Serious	Serious	Moderate	Low	Moderate	Moderate	Moderate	Serious
Dickens (2014)	Moderate	Moderate	Moderate	Low	Serious	Moderate	Moderate	Serious
Dix-Peek (2023)	Serious	Moderate	Moderate	Moderate	Moderate	Low	Moderate	Serious
Eber-Schulz (2018)	Moderate	Moderate	Low	Low	Moderate	Low	Moderate	Moderate
Ekpe (2019)	Serious	Moderate	Low	Low	Moderate	Low	Moderate	Serious
Elenwo et al. (2021)	Moderate	Serious	Moderate	Low	Moderate	Moderate	Moderate	Serious
Elmore (2021)	Moderate	Moderate	Low	Low	Moderate	Moderate	Moderate	Moderate
Feleke (2022)	Moderate	Moderate	Low	Low	Moderate	Low	Moderate	Moderate
Fitzpatrick et al. (2019)	Serious	Serious	Low	Low	Moderate	Low	Moderate	Serious
Forae et al. (2014)	Serious	Serious	Low	Low	Moderate	Low	Moderate	Serious
Galukande (2015)	Serious	Moderate	Moderate	Low	Moderate	Moderate	Moderate	Serious
Galukande (2015)	Serious	Serious	Low	Low	Moderate	Low	Moderate	Serious
Gebrehiwot (2019)	Serious	Serious	Moderate	Low	Moderate	Low	Moderate	Serious
Gebremariam (2023)	Moderate	Moderate	Low	Low	Low	Moderate	Moderate	Moderate
Gebretsadik (2021)	Serious	Moderate	Moderate	Low	Moderate	Moderate	Moderate	Serious
Gnanamuttupulle (2021)	Serious	Moderate	Low	Low	Moderate	Low	Moderate	Serious
Groenewald (2019)	Serious	Moderate	Moderate	Low	Moderate	Moderate	Moderate	Serious
Hadgu (2018)	Serious	Moderate	Low	Low	Moderate	Low	Moderate	Serious
Hafiz et al. (2018)	Serious	Serious	Low	Low	Moderate	Moderate	Moderate	Serious
Hassan (2017)	Serious	Moderate	Moderate	Low	Moderate	Moderate	Moderate	Serious
Heunis (2018)	Serious	Moderate	Moderate	Moderate	Moderate	Moderate	Moderate	Serious
Ikeri et al. (2018)	Serious	Serious	Moderate	Low	Moderate	Moderate	Moderate	Serious
Jedy-Agba et al. (2017)	Moderate	Moderate	Low	Low	Moderate	Low	Moderate	Moderate
Kakudji (2020)	Serious	Moderate	Moderate	Low	Moderate	Moderate	Moderate	Serious
Kakudji (2021)	Serious	Moderate	Moderate	Low	Moderate	Moderate	Moderate	Serious
Kantelhardt (2014)	Moderate	Moderate	Moderate	Low	Moderate	Low	Moderate	Moderate
Khan (2022)	Serious	Serious	Moderate	Moderate	Moderate	Moderate	Moderate	Serious
Knapp et al. (2021)	Moderate	Moderate	Low	Moderate	Moderate	Low	Moderate	Moderate

Langenhoven (2016)	Serious	Moderate	Moderate	Moderate	Serious	Moderate	Moderate	Serious
Lopes et al. (2015)	Serious	Moderate	Moderate	Low	Moderate	Moderate	Moderate	Serious
Lupicki (2018)	Serious	Moderate	Moderate	Low	Moderate	Moderate	Moderate	Serious
Mannell (2020)	Moderate	Moderate	Low	Low	Moderate	Low	Moderate	Moderate
Mansouri (2019)	Serious	Serious	Moderate	Low	Moderate	Moderate	Moderate	Serious
Mapanga (2023)	Moderate	Moderate	Low	Low	Moderate	Low	Moderate	Moderate
Martei (2023)	Serious	Serious	Moderate	Low	Low	Moderate	Moderate	Serious
Matheka (2023)	Serious	Moderate	Low	Low	Moderate	Low	Moderate	Serious
Mavhungu (2021)	Serious	Moderate	Moderate	Moderate	Serious	Moderate	Moderate	Serious
Menon (2017)	Moderate	Moderate	Low	Low	Moderate	Moderate	Moderate	Moderate
Miguel et al. (2017)	Serious	Moderate	Low	Low	Moderate	Low	Moderate	Serious
Misganaw (2023)	Moderate	Moderate	Low	Low	Moderate	Low	Moderate	Moderate
Moodley (2018)	Serious	Moderate	Moderate	Moderate	Moderate	Moderate	Moderate	Serious
Mthembu (2021)	Serious	Moderate	Low	Moderate	Serious	Moderate	Moderate	Serious
Murugan (2014)	Moderate	Moderate	Moderate	Low	Serious	Moderate	Moderate	Serious
Mushonga (2020)	Serious	Serious	Moderate	Low	Serious	Moderate	Moderate	Serious
Mvila et al. (2014)	Serious	Serious	Moderate	Low	Moderate	Moderate	Moderate	Serious
Mwakigonja et al (2016)	Serious	Moderate	Moderate	Low	Moderate	Low	Moderate	Serious
Mwakigonja et al (2017)	Serious	Moderate	Low	Low	Moderate	Low	Moderate	Serious
Ndiaye et al. (2020)	Serious	Serious	Moderate	Low	Moderate	Moderate	Moderate	Serious
Ngidi (2017)	Serious	Moderate	Low	Low	Moderate	Moderate	Moderate	Serious
Nsaful et al. (2020)	Serious	Serious	Moderate	Low	Moderate	Moderate	Moderate	Serious
Ntirenganya (2022)	Moderate	Moderate	Low	Low	Serious	Low	Moderate	Serious
Okifo et al. (2021)	Serious	Serious	Low	Low	Moderate	Moderate	Moderate	Serious
Olasehinde et al (2021)	Moderate	Serious	Low	Low	Moderate	Low	Moderate	Serious
Oloagun et al. (2020)	Serious	Serious	Low	Low	Moderate	Moderate	Moderate	Serious
Pace (2016)	Serious	Moderate	Low	Low	Moderate	Moderate	Moderate	Serious
Pace (2023)	Serious	Moderate	Moderate	Moderate	Moderate	Moderate	Moderate	Serious
Pumpalova (2022)	Moderate	Serious	Low	Moderate	Moderate	Low	Moderate	Serious
Rambau (2014)	Serious	Moderate	Low	Low	Moderate	Low	Moderate	Serious
Ranaivomanana (2021)	Serious	Moderate	Moderate	Low	Moderate	Moderate	Moderate	Serious
Rapoport (2022)	Serious	Moderate	Moderate	Low	Moderate	Moderate	Moderate	Serious
Rayne (2019)	Serious	Moderate	Low	Low	Moderate	Moderate	Moderate	Serious
Rayne (2019)	Serious	Moderate	Low	Low	Moderate	Moderate	Moderate	Serious
Ruff (2018)	Moderate	Moderate	Low	Low	Moderate	Moderate	Moderate	Moderate
Rweyemamu (2021)	Serious	Serious	Moderate	Low	Moderate	Moderate	Moderate	Serious
Rweyemamu (2021)	Serious	Serious	Moderate	Low	Moderate	Low	Moderate	Serious
Sayed (2014)	Serious	Moderate	Low	Low	Moderate	Low	Moderate	Serious
Scherber et al. (2014)	Moderate	Serious	Moderate	Low	Serious	Moderate	Moderate	Serious
Schleimer (2019)	Serious	Moderate	Low	Moderate	Serious	Moderate	Moderate	Serious
Shiferaw (2020)	Moderate	Moderate	Low	Low	Moderate	Low	Moderate	Moderate
Shita (2022)	Serious	Moderate	Low	Low	Serious	Moderate	Moderate	Serious
Somé et al. (2022)	Serious	Moderate	Low	Moderate	Moderate	Low	Moderate	Serious
Songiso (2020)	Serious	Moderate	Low	Low	Moderate	Low	Moderate	Serious
Ssentongo et al. (2022)	Serious	Serious	Moderate	Low	Moderate	Moderate	Moderate	Serious
Tesfaw (2021)	Moderate	Moderate	Moderate	Low	Moderate	Low	Moderate	Moderate
Torrorey-Sawe (2020)	Moderate	Moderate	Low	Low	Moderate	Low	Moderate	Moderate
Traore et al (2015)	Serious	Serious	Moderate	Low	Moderate	Moderate	Moderate	Serious
Traore et al. (2021)	Serious	Serious	Moderate	Low	Moderate	Moderate	Moderate	Serious
Tuwei (2021)	Serious	Moderate	Moderate	Low	Serious	Moderate	Moderate	Serious

Wambua (2022)	Serious	Moderate	Moderate	Low	Moderate	Moderate	Moderate	Serious
Wuraola et al. (2023)	Moderate	Serious	Low	Low	Moderate	Low	Moderate	Serious
Youngblood (2020)	Moderate	Moderate	Low	Low	Moderate	Low	Moderate	Moderate
Zheng et al. (2018)	Moderate	Moderate	Low	Low	Moderate	Low	Moderate	Moderate
Zongo et al. (2016)	Serious	Serious	Moderate	Low	Moderate	Moderate	Moderate	Serious
Zuze (2018)	Serious	Moderate	Moderate	Moderate	Serious	Moderate	Moderate	Serious
Anyigba et al. (2025)	Moderate	Moderate	Low	Low	Moderate	Moderate	Moderate	Moderate
Assele et al. (2025)	Moderate	Moderate	Low	Low	Moderate	Low–moderate	Moderate	Moderate
Ballé et al. PAM50 (2024)	Moderate	Serious	Low	Low	Moderate	Low	Moderate	Serious
Bhangdia et al. (2025)	Moderate	Moderate	Low	Low	Moderate	Moderate	Moderate	Moderate
Borges et al. (2025)	Moderate	Moderate	Low	Low	Moderate	Low–moderate	Moderate	Moderate
Chibatamoto et al. (2025)	Moderate	Moderate	Low	Low	Moderate	Moderate	Moderate	Moderate
Cummings-John et al. (2025)	Moderate	Moderate–serious	Low	Low	Moderate	Moderate	Moderate	Moderate –serious
Dedey et al. (2024)	Moderate	Moderate	Low	Low	Moderate	Moderate	Moderate	Moderate
Ekdahl Hjelm et al. (2025)	Moderate	Serious	Low	Low	Moderate	Low	Moderate	Serious
Gnangnon et al. (2024)	Moderate	Moderate	Low	Low	Moderate	Low–moderate	Moderate	Moderate
Kara et al. (2025)	Moderate	Serious	Low	Low	Moderate	Low–moderate	Moderate	Serious
Kivuyo et al. surgical margins (2025)	Moderate	Serious	Low	Low	Moderate	Low–moderate	Moderate	Serious
Kivuyo et al. treatment outcomes (2025)	Moderate	Moderate	Low	Low	Moderate	Low–moderate	Moderate	Moderate
Kretzmann & Adeniyi (2025)	Moderate	Moderate	Low	Low	Moderate	Low	Moderate	Moderate
Mensah et al. (2025)	Moderate	Moderate	Low	Low	Moderate	Moderate	Moderate	Moderate
Morgan et al. (2025)	Moderate	Moderate	Low	Low	Moderate	Moderate	Moderate	Moderate
Narh et al. (2025)	Moderate	Moderate	Low	Low	Moderate	Moderate	Moderate	Moderate
Pat et al. (2025)	Moderate	Moderate	Low	Low	Moderate	Low–moderate	Moderate	Moderate
Shewarega et al. (2025)	Moderate	Serious	Low	Low	Moderate	Moderate	Moderate	Serious
Songiso et al. (2024)	Moderate	Moderate	Low	Low	Moderate	Low–moderate	Moderate	Moderate
Tambe et al. (2025)	Serious	Serious	Moderate	Low	Serious	Moderate–serious	Serious	Serious
