# Supplementary material for: Stage at Diagnosis and Molecular Subtype Distribution of Breast Cancer in Sub‐Saharan Africa: A Systematic Review
Source: Cancer Rep (Hoboken). 2026 Jun 10;9(6):e70594. doi: 10.1002/cnr2.70594 (PMC13250637; doi:10.1002/cnr2.70594)
Supplement: Supplementary file 4 — Table S3: Characteristics of the studies included in this study (country, study design, study period, stages of breast cancer reported and age). [file CNR2-9-e70594-s003.pdf]

Supplementary Table 3: Characteristics of the studies included in this study (country, study design, study period, stages of breast cancer reported and age).

REFERENCE	Country	Study design	Study period	Staged patients (n)	I	I/II	II	III	IV	III/IV	Mean age (years)
Bambara et al (2017)	BURKINA FASO	Cross-sectional	2015-2016	80	5	0	35	23	17	0	48,2
Somé et al (2022)	BURKINA FASO	Retrospective cohort	2015 - 2021	313	30	0	0	149	134	0	46,6
Dedey et al (2016)	GHANA	Retrospective cohort	2013	179	5	0	47	125	2	0	51,1
Okifo et al (2021)	GHANA	Retrospective cohort	2011-2019	188	5	0	34	77	72	0	49,9
Scherber et al (2014)	GHANA	Retrospective cohort	2008 – 2011	463	8	0	72	347	36	0	50,8
Ssentongo et al (2022)	GHANA	Retrospective cohort	2014 - 2017	130	6	0	46	78	0	0	51

**Nsaful et al (2020)**	GHANA	Case series	2016-2019	4	0	0	0	2	2	0	32,5
**Traore et al (2021)**	Guinea Conakry	Retrospective cohort	2007 to 2016	113	0	18	0	92	3	0	48,2
**Traore et al (2015)**	Guinea Conakry	Retrospective cohort	2007 to 2016	55	0	16	0	0	0	39	48,3
**Diakite et al (2020)**	MALI	Case control	2018-2019	60	0	0	60	0	0	0	43,72
**Brahim et al (2022)**	MAURITANIA	Retrospective cohort	2009-2020	540	7	0	167	242	124	0	39
**Ayandipo et al (2023)**	NIGERIA	Longitudinal cohort	2009-2013	67	25	0	42	0	0	0	43
**Elenwo et al (2021)**	NIGERIA	Retrospective cohort	2016-2019	61	2	0	17	34	8	0	41
**Forae et al (2014)**	NIGERIA	Retrospective cohort	2005-2011	261	12	0	50	98	101	0	46
**Akingbade et al (2022)**	NIGERIA	Qualitative study	Not reported	27	7	0	5	11	4	0	48

**Hafiz et al (2018)**	NIGERIA	Prospective cohort	2015	105	4	0	38	52	11	0	48,1
**Ikeri et al (2018)**	NIGERIA	Retrospective cohort	2010 - 2017	51	0	0	12	39	0	0	49
**Jedy-Agba et al (2017)**	NIGERIA	Multicentre Case-control	2014-2016	300	5	0	92	157	46	0	45,4
**Knapp et al (2021)**	NIGERIA	Prospective cohort breast cancer database	2009-2019	586	8	0	88	355	135	0	49
**Oloagun et al (2020)**	NIGERIA	Retrospective cohort	2011-2015	82	1	0	9	61	11	0	48.9
**Olasehinde et al (2021)**	NIGERIA	Retrospective cohortuntil 2016 pro 2016	2010 - 2018	591	7	0	92	369	123	0	49,8
**Wuraola et al (2023)**	NIGERIA	Prospective cohort	2019 – 2021	153	2	0	36	108	7	0	47
**Zheng et al (2018)**	NIGERIA	Case-control	1998-2014	577	6	0	74	241	256	0	47,5

**Adeoluwa et al (2020)**	NIGERIA	Prospective cohort	2017 - 2019	251	2	0	35	168	46	0	46,1
**Ali-Gombe et al (2021)**	NIGERIA	Retrospective Cross-sectional	2004-2008	351	15	0	99	209	28	0	47,6
**Fitzpatrick et al (2019)**	SENEGAL	Retrospective cohort	2001-2006	194	4	0	32	81	77	0	46
**Ndiaye et al (2020)**	SENEGAL	Case control	2019	17	1	0	10	6	0	0	39,5
**Zongo et al (2016)**	Senegal	Case series	2010-2011	3	0	0	0	1	2	0	26,6
**Ablavi-Ife et al (2023)**	TOGO	Retrospective cohort	2016-2020	117	3	0	45	42	27	0	52,05
**Darre et al (2021)**	TOGO	Retrospective cohort	2000-2019	490	0	0	383	107	0	0	46,7
**Darre et al (2023)**	TOGO	Prospective cohort	2021	62	0	0	0	43	19	0	30,9
**Hassan (2017)**	DJIBOUTI	Prospective cohort	2012 - 2017	102	7	0	37	34	24	0	48

**Bacha (2021)**	ETHIOPIA	Retrospective cohort	2015-2020	642	26	0	210	229	177	0	NR
**Eber-Schulz (2018)**	ETHIOPIA	Prospective cohort	2010 -2016	102	0	29	0	66	7	0	45
**Feleke (2022)**	ETHIOPIA	Retrospective cohort	2018 - 2022	322	58	0	88	83	93	0	43,8
**Gebrehiwot (2019)**	ETHIOPIA	Case-control	2015 – 2016	95	19	0	23	25	28	0	40,5
**Gebremariam (2023)**	ETHIOPIA	Prospective cohort	2017 - 2018	404	0	145	0	0	0	259	44,4
**Gebretsadik (2021)**	ETHIOPIA	Retrospective cohort	2013 – 2019	475	21	0	82	192	180	0	38
**Hadgu (2018)**	ETHIOPIA	Cross-sectional	2012 - 2015	96	19	0	37	36	4	0	43
**Misganaw (2023)**	ETHIOPIA	Retrospective cohort	2015 - 2018	412	15	0	98	156	143	0	45

**Shita (2022)**	ETHIOPIA	Retrospective cohort	2013 - 2018	302	0	50	0	161	91	0	39
**Kantelhardt (2014)**	ETHIOPIA	Retrospective cohort	2005-2010	787	0	188	0	456	143	0	42
**Shiferaw (2020)**	ETHIOPIA	Retrospective cohort	2018	362	28	0	16	318	0	0	44,32
**Tesfaw (2021)**	ETHIOPIA	Cross-sectional	2019-2020	321	37	0	70	211	3	0	40
**Akinyi et al (2019)**	KENYA	Cross-sectional	2019	79	8	0	33	38	0	0	48
**Brand (2017)**	KENYA	Retrospective cohort	2008-2017	119	73	0	46	0	0	0	54,5
**Ekpe (2019)**	KENYA	Retrospective cohort	2012 -2018	125	0	0	40	45	40	0	47
**Sayed (2014)**	KENYA	Retrospective cohort	2011-2012	99	12	0	26	43	18	0	47,5
**Torrorey-Sawe (2020)**	KENYA	Prospective cohort	2013 - 2016	48	5	0	22	17	4	0	46,9
**Tuwei (2021)**	KENYA	Retrospective cohort	2015-2019	50	1	0	17	14	18	0	45,44

**Wambua (2022)**	KENYA	Retrospective cohort	2015-2020	110	9	0	23	49	29	0	56
**Matheka (2023)**	KENYA	Retrospective cohort	2009-2017	131	0	0	43	48	40	0	47
**Ranaivomanana (2021)**	MADAGASCAR	Retrospective cohort	2011 - 2018	62	0	8	0	34	20	0	52,83
**Youngblood (2020)**	MALAWI	Prospective cohort	2016-2018	91	0	0	16	48	27	0	49,2
**Zuze (2018)**	MALAWI	Conference abstract	2016-2018	54	0	0	0	0	0	54	48
**Ntirenganya (2022)**	RWANDA	Retrospective cohort	2022	340	21	0	156	125	38	0	49
**Pace (2016)**	RWANDA	Retrospective cohort (BUTARO)	2012-2013	164	0	35	0	77	52	0	41
**Pace (2023)**	RWANDA	Retrospective cohort	2018 – 2019	29	0	6	0	0	0	23	55
**Schleimer (2019)**	RWANDA	Retrospective cohort	2014 – 2015	142	3	0	37	57	45	0	54

**Achan (2023)**	UGANDA	Cross-sectional (Mulago)	2019	402	15	0	79	168	140	0	47,1
**Galukande (2015)**	UGANDA	Retrospective cohort (Mulago)	2004-2007 2010-2012	209	5	0	17	152	35	0	45
**Menon (2017)**	UGANDA	Retrospective cohort	2003-2010	194	7	0	6	129	52	0	45
**Rweyemamu (2021)**	TANZANIA	Cross-sectional	2019-2021	72	1	0	9	42	20	0	47,99
**Gnanamuttupulle (2021)**	TANZANIA	Cross-sectional	2018-2019	116	0	22	0	0	0	94	53
**Mwakigonja et al (2016)**	TANZANIA	Prospective cohort	2011-2012	86	0	0	14	72	0	0	52,1
**Rambau (2014)**	TANZANIA	Retrospective cohort	2014	52	3	0	7	36	6	0	49
**Rweyemamu (2021)**	TANZANIA	Cross-sectional	2019-2020	263	4	0	41	146	72	0	44,49
**Mansouri (2019)**	TANZANIA	Retrospective cohort	2016-2017	74	3	0	7	36	28	0	51,32

**Mwakigonja et al (2017)**	TANZANIA	Cross-sectional	2013	50	1	0	10	39	0	0	48,36
**Songiso (2020)**	ZAMBIA	Prospective cohort	2018-2019	112	0	42	0	62	8	0	NR
**Elmore (2021)**	ZIMBABWE	Retrospective cohort	2014-2018	217	0	0	11	54	152	0	51,3
**Mushonga (2020)**	ZIMBABWE	Retrospective cohort	2014-2016	75	1	0	7	29	38	0	52
**Lopes et al (2015)**	ANGOLA	Retrospective cohort	2006-2014	1323	65	0	225	998	35	0	47
**Miguel et al (2017)**	ANGOLA	Prospective cohort	2011-2014	132	1	0	35	91	5	0	47
**Mvila et al (2014)**	DR CONGO	Prospective cohort	2010-2012	86	2	0	19	65	0	0	NR
**Bhatia (2019)**	BOTSWANA	Retrospective cohort	2011-2015	51	2	0	14	35	0	0	54
**Wester (2022)**	BOTSWANA	Retrospective	2010-2020	609	23	0	163	343	80	0	53
**Martei (2023)**	BOTSWANA	Qualitative study	2020-2020	30	0	0	8	22	0	0	NR
**Rayne (2019)**	SOUTH AFRICA	Cross-sectional	NR	231	33	0	63	129	6	0	56

**Cubasch (2017)**	SOUTH AFRICA	Retrospective cohort	2009 - 2011	491	32	0	262	151	46	0	NR
**Dix-Peek (2023)**	SOUTH AFRICA	Case control	2014-2020	994	38	0	378	439	139	0	53
**Groenewald (2019)**	SOUTH AFRICA	Retrospective cohort	2013 – 2015	505	46	0	249	189	21	0	53,6
**Khan (2022)**	SOUTH AFRICA	Retrospective cohort	2011 - 2012	41	4	0	34	3	0	0	58,4
**Langenhoven (2016)**	SOUTH AFRICA	Case control	2010-2011	586	32	0	231	219	104	0	56
**Lupicki (2018)**	SOUTH AFRICA	Retrospective cohort	2006 - 2010	30	2	0	7	21	0	0	48,2
**Mavhungu (2021)**	SOUTH AFRICA	Retrospective cohort	2015-2017	248	0	78	0	0	0	170	55
**Moodley (2018)**	SOUTH AFRICA	cross-sectional study	2015 - 2016	187	0	144	0	0	0	43	NR
**Mthembu (2021)**	SOUTH AFRICA	Retrospective cohort cross-	2020-2021	220	0	83	0	0	0	137	54,8

		sectional descriptive									
**Murugan (2014)**	SOUTH AFRICA	Retrospective cohort	2006-2012	1051	53	0	431	480	87	0	55
**Ngidi (2017)**	SOUTH AFRICA	Retrospective cohort	2012 - 2015	65	0	0	8	55	2	0	48,5
**Rapoport (2022)**	SOUTH AFRICA	Case-control	published 2022	98	13	0	71	14	0	0	52
**Rayne (2019)**	SOUTH AFRICA	(SABCHO) cohort study	2016-2017	232	36	0	66	130	0	0	55
**Ruff (2018)**	SOUTH AFRICA	Retrospective cohort	2009-2011	554	32	0	273	249	0	0	52
**Chilwesa (2020)**	SOUTH AFRICA	Prospective cohort	2017	41	0	0	0	41	0	0	51,5
**Ayeni (2023)**	SOUTH AFRICA	(SABCHO) cohort study	2015-2019	3261	0	1353	0	1339	569	0	56,9
**Bhuiyan (2022)**	SOUTH AFRICA	Prospective cohort	2020-2021	274	0	67	0	0	0	207	54
**Čačala (2021)**	SOUTH AFRICA	Retrospective cohort	2002-2007 2015	435	0	0	14	248	173	0	52,6

		Prospective cohort	- 2018								
**Cubasch (2018)**	SOUTH AFRICA	Retrospective cohort	2009 – 2011	602	31	0	276	245	50	0	54,4
**Dickens (2014)**	SOUTH AFRICA	Prospective cohort	2006 – 2012	1051	53	0	431	480	87	0	55
**Heunis (2018)**	SOUTH AFRICA	Retrospective cohort	2010 – 2014	657	33	0	337	287	0	0	NR
**Kakudji (2021)**	SOUTH AFRICA	Cross-sectional	2012-2018	116	11	0	33	42	30	0	56,35
**Kakudji (2020)**	SOUTH AFRICA	Retrospective cohort	2012-2018	138	15	0	39	46	38	0	NR
**Mannell (2020)**	SOUTH AFRICA	Prospective cohort	2017-2018	112	0	0	7	78	27	0	54
**Mapanga (2023)**	SOUTH AFRICA	Prospective cohort	2015 -2019	3497	0	1439	0	0	0	2058	55,7
**Pumpalova (2022)**	SOUTH AFRICA	Prospective cohort	2015 - 2019	1187	40	0	335	200	612	0	NR

**Boucheron (2021)**	Namibia 477 Uganda 418 Nigeria 383 Zambia 198	Multicenter cohort (ABCDO)	2014 - 2017	1381	0	N121<br>U96<br>NI39<br>Z19	0	N29<br>0<br>U22<br>6<br>NI2<br>56Z<br>131	N66<br>U64<br>NI6<br>0<br>Z13	0	50,3
**Anyigba et al. (2025)**	Ghana	Cohort study / Ghana BCOPGh	Dec 2018 –Mar 2023	47	9	0	29	7	2	0	NR
**Assele et al. (2025)**	Ethiopia	Hospital-based retrospective cohort	Jan 2020 –Dec 2022	492	73	0	105	138	176	0	NR
**Ballé et al. PAM50 (2024)**	Ethiopia	Prospective observational study	2010-2018	76	20	0	1	49	6	0	NR
**Bhangdia et al. (2025)**	Rwanda	Retrospective cohort	2012 – 2016	426	0	106	0	0	0	320	49
**Borges et al. (2025)**	Cape Verde	Retrospective study	2000 –May 2024	584	127	0	151	231	75	0	52.1
**Chibatamoto et al. (2025)**	Botswana	Cross-sectional hospital-based survey	15 Sep– 15 Dec 2023	186	14	0	82	81	9	0	52

**Cummings-John et al. (2025)**	Sierra Leone	Retrospective cross-sectional	2018–2021	228	0	0	24	169	35	0	47 ± 14
**Dedey et al. (2024)**	Ghana	Cross-sectional health-seeking study	Jan–Dec 2022	589	0	229	0	0	0	360	52.6 ± 12.1
**Ekdahl Hjelm et al. (2025)**	Ethiopia	Prospective Ethiopian cohort with Swedish comparator	2021-2023	92	3	0	31	41	17	0	33.3
**Gnangnon et al. (2024)**	Benin	Hospital-based retrospective study	2014–2020	284	17	0	62	135	70	0	48.7
**Kara et al. (2025)**	South Africa	Retrospective cohort review	Jan 2019–Dec 2021	1214	372	0	718	124	0	0	32.2
**Kivuyo et al. surgical margins (2025)**	Tanzania	Retrospective cohort	2018-2024	614	25	0	161	428	0	0	NR
**Kivuyo et al. treatment outcomes (2025)**	Tanzania	Retrospective cohort	2022	298	0	0	52	196	50	0	53.2 ± 13.6
**Kretzmann & Adeniyi (2025)**	South Africa	Retrospective cross-sectional record review	2022–2023	278	19	0	92	134	33	0	58.7

**Mensah et al. (2025)**	Ghana	GBCI KPI/diagnosis management study	2021	213	0	0	22	107	84	0	NR
**Morgan et al. (2025)**	Malawi	Prospective cohort	Dec 2016 –Oct 2018	91	0	0	13	54	24	0	50
**Narh et al. (2025)**	Ghana	Prospective care-pathway cohort	Mar 2023 –Feb 2024	102	17	0	23	30	32	0	NR
**Pat et al. (2025)**	Mauritius (EAST)	Retrospective cohort	2017 – 2020	1059	470	0	213	269	107	0	NR
**Shewarega et al. (2025)**	Ethiopia	Cross-sectional study	Oct–Nov 2021	269	0	89	0	0	0	180	44
**Songiso et al. (2024)**	Zambia	Prospective cohort / one-stop clinic	2018-2022	302	0	132	0	138	32	0	NR
**Tambe et al. (2025)**	Cameroon	Hospital registry/proceedings abstract	2020-2025	271	17	0	98	118	38	0	47
**Anyigba et al. (2025)**	Ghana	Cohort study / Ghana BCOPGh	Dec 2018 –Mar 2023	47	9	0	29	7	2	0	NR
